# A Data‐Driven Simplified Nernst Equation for Estimating Reduction Potentials in Groundwater from pH and Temperature

**DOI:** 10.1111/gwat.70010

**Published:** 2025-08-07

**Authors:** Gordon Bowman, Gabe Harris, Matthew Kirk, Qusheng Jin

**Affiliations:** ^1^ Department of Earth Science University of Oregon Eugene OR 97403; ^2^ Department of Geology Kansas State University Manhattan KS 66506

## Abstract

Reduction potentials of redox couples are fundamental for understanding subsurface geochemistry and guiding water resource exploration and management. Reduction potentials are routinely calculated with the Nernst equation, which requires detailed chemical composition data and complex speciation modeling—factors that limit its application in large‐scale or data‐limited field settings. To address these limitations, we developed a data‐driven simplified Nernst equation that estimates the reduction potentials of individual redox couples using only pH and temperature. By integrating geochemical modeling with a global groundwater chemistry dataset, we demonstrate that pH is the dominant control on redox potential, while temperature and redox species activity play secondary roles. The resulting formulation reduces computational demands while maintaining high‐predictive accuracy across diverse groundwater environments. This approach enables rapid and scalable estimation of reduction potentials, supporting applications in geochemical modeling, contaminant transport prediction, and groundwater quality assessments. Furthermore, it offers a thermodynamically grounded yet practical framework for interpreting electron transfer dynamics in natural groundwater systems.

## Introduction

Reduction potentials of individual redox couples are essential for assessing the redox state of natural waters, influencing processes such as contaminant transport, biogeochemical cycling, and water quality management (Burgin and Loecke 2023; Linnik et al. 2023). These potentials indicate the thermodynamic favorability and directionality of electron transfer reactions by quantifying the tendency of chemical species to accept or donate electrons. Alongside pH, reduction potential is one of the most influential parameters controlling the chemical speciation and mobility of elements in aqueous environments (Stumm and Morgan 1996). As such, accurate reduction potential assessment is essential for both modeling and field applications of water resource exploration and management, informing tasks from assessing water quality and forecasting pollutant migration to developing sustainable remediation and resource management strategies (Christensen et al. 2000; Meckenstock et al. 2015; Wang et al. 2023).

The redox state of natural waters is governed by the presence and reactivity of redox couples—pairs of electron acceptors and their corresponding reduced forms. In groundwater systems, common electron acceptors include dissolved oxygen (DO), nitrate, Mn(IV) oxides such as pyrolusite (β‐MnO_2_) and birnessite (δ‐MnO_2_), Fe(III) oxyhydroxides such as ferrihydrite (Fe(OH)_3_), goethite (FeOOH), and hematite (Fe_2_O_3_), as well as sulfate and bicarbonate (Bethke et al. 2011; Jin and Kirk 2018a). The reduction potential of a redox couple is defined in terms of its half‐cell reduction reaction,

(1) $$\nu_{A}A+\nu_{H}H^{+}+ze^{-}\to v_{A^{-}}A^{-}$$

where A and A^−^ are an electron acceptor and its reduced form, respectively, *ν*_A_ and $v_{A^{-}}$ are their stoichiometric coefficients, *ν*_H_ is the number of protons consumed per reaction, and *z* is the number of transferred electrons (see Table 1). The reduction potential Eh (V) is calculated using the Nernst equation:

(2) $$\mathrm{Eh}={\mathrm{Eh}}^{o}-\frac{\mathrm{RT}}{\mathrm{zF}}\left(L_{10}\nu_{H}\mathrm{pH}+\ln Q_{A}\right)$$

Here Eh^o^ is the standard potential (V) at temperature *T* in Kelvin (or K), *L*_10_ represents ln(10), *R* is the gas constant (J·mol^−1^·K^−1^), *F* is Faraday's constant (C·mol^−1^), and *Q*_A_ is the activity product of the redox couple, that is, the ratio in chemical activity of A^−^ to A.

**Table 1:** Half‐Reactions of Electron Acceptor Reduction and Corresponding Pearson's Correlation Coefficients between Reduction Potentials Calculated Using the Nernst Equation and Electrode Potentials Directly Measured in the Field, along with Correlations between Calculated Potentials and Temperature *T*, pH, or the Product of pH and *T*.

Half‐Reaction	Pearson's Coefficient		Pearson's Coefficient			
	*n* ^a^	Electrode Eh	*n*	*T*	pH	pH·*T*
$O_{2}\left(\mathrm{aq}\right)+4H^{+}+4e^{-}\to 2H_{2}O$	992	0.19 (<0.001)^b^	31,871	−0.25 (<0.001)	−0.98 (<0.001)	−0.99 (<0.001)
${\mathrm{NO}}_{3}^{-}+6H^{+}+5e^{-}\to \frac{1}{2}N_{2}\left(\mathrm{aq}\right)+3H_{2}O$	6	n.d.^c^	20	n.d.	n.d.	n.d.
${\mathrm{NO}}_{3}^{-}+10H^{+}+8e^{-}\to {\mathrm{NH}}_{4}^{+}+3H_{2}O$	291	−0.12 (0.040)	8623	−0.27 (<0.001)	−0.99 (<0.001)	−0.99 (<0.001)
$\beta ‐{\mathrm{MnO}}_{2}+4H^{+}+2e^{-}\to {\mathrm{Mn}}^{2+}+2H_{2}O$	923	0.25 (<0.001)	38,966	−0.12 (<0.001)	−0.98 (<0.001)	−0.98 (<0.001)
${\mathrm{Mn}}_{8}O_{14}\cdot 5H_{2}O+28H^{+}+12e^{-}\to 8{\mathrm{Mn}}^{2+}+19H_{2}O$	923	0.25 (<0.001)	38,966	−0.11 (<0.001)	−0.97 (<0.001)	−0.97 (<0.001)
$\text{FeOOH}+3H^{+}+e^{-}\to {\mathrm{Fe}}^{2+}+2H_{2}O$	145	0.26 (0.002)	8364	−0.20 (<0.001)	−0.96 (<0.001)	−0.95 (<0.001)
$\mathrm{Fe}{\left(\mathrm{OH}\right)}_{3}+3H^{+}+e^{-}\to {\mathrm{Fe}}^{2+}+3H_{2}O$	145	0.26 (0.002)	8364	−0.30 (<0.001)	−0.96 (<0.001)	−0.97 (<0.001)
$\frac{1}{2}{\mathrm{Fe}}_{2}O_{3}+3H^{+}+e^{-}\to {\mathrm{Fe}}^{2+}+\frac{3}{2}H_{2}O$	145	0.26 (0.002)	8364	−0.32 (<0.001)	−0.96 (<0.001)	−0.97 (<0.001)
${\mathrm{SO}}_{4}^{2-}+10H^{+}+8e^{-}\to H_{2}S\left(\mathrm{aq}\right)+4H_{2}O$	40	0.11 (0.514)	814	−0.14 (<0.001)	−0.98 (<0.001)	−0.99 (<0.001)
${\mathrm{HCO}}_{3}^{-}+9H^{+}+8e^{-}\to {\mathrm{CH}}_{4}\left(\mathrm{aq}\right)+3H_{2}O$	6	n.d.	239	−0.17 (0.010)	−0.98 (<0.001)	−0.99 (<0.001)

^a^ Number of data points.

^b^ Values in parentheses are *p*‐values.

^c^ n.d., not determined.

The Nernst equation is theoretically straightforward, but its rigorous application to groundwater systems is complicated by their non‐ideal thermodynamic behavior. Groundwater chemistry is shaped by complex interactions among solutes, mineral surfaces, and microbial processes, requiring comprehensive speciation modeling to estimate activity coefficients and redox species concentrations accurately. These treatments depend on high‐quality water chemistry data and strict enforcement of charge balance to reliably compute key parameters, such as ionic strength and the activities of redox species (Bethke 2022).

Additionally, the redox state of natural waters is often assessed in situ using potentiometric techniques that measure the potential difference between a sensing electrode (typically platinum) and a reference electrode (e.g., Ag/AgCl). However, the resulting electrode potentials frequently diverge from reduction potentials calculated via the Nernst equation for individual redox couples (Lindberg and Runnells 1984; Stefánsson et al. 2005). This discrepancy arises because natural waters commonly contain multiple redox species that are not in mutual equilibrium. As a result, the redox conditions of such systems cannot be reliably represented by a single Eh value (Sigg 2000; Nordstrom and Wilde 2005). These limitations complicate the interpretation of electrode measurements and hinder their application in large‐scale hydrogeochemical modeling and groundwater quality assessments.

Given these challenges, studies have demonstrated that pH serves as the dominant control on reduction potentials in groundwater systems, whereas temperature and redox species activity play secondary roles (Bethke et al. 2011; Jin and Kirk 2018a). This underscores the potential value of a simplified, pH‐based approach for estimating reduction potentials as a practical alternative to detailed speciation modeling. However, a robust, generalizable equation for predicting reduction potentials across diverse hydrogeochemical settings has yet to be developed.

In this study, we integrate geochemical speciation modeling with big data analytics (Haddad et al. 2013; Yang et al. 2019) and derive a simplified Nernst equation that predicts reduction potentials of individual redox couples as a function of pH. By analyzing a large dataset encompassing diverse groundwater chemical compositions, we assess the relative influence of pH, temperature, and redox species activity on reduction potentials calculated from the Nernst equation. Our approach reduces computational complexity while maintaining accuracy in estimating reduction potentials of individual redox couples, offering a scalable and efficient tool for groundwater monitoring, contaminant transport modeling, and biogeochemical assessment. Furthermore, our findings provide new insights into the thermodynamic patterns of groundwater redox reactions, improving the conceptual framework for redox ladder applications in subsurface environments.

## Materials and Methods

### Groundwater Dataset and Data Quality Control

We assembled a high‐quality dataset of groundwater chemical composition in order to construct a predictive model for the reduction potentials of individual redox couples in groundwater. The dataset was sourced from two databases: the USGS National Water Information System (NWIS) (U.S. Geological Survey 2016) and the Global Freshwater Quality Database (GEMStat) (UN‐GEMS Water 2023). The NWIS database provided 362,424 datapoints from the United States, with records dating from 1948 to January 2023, while the GEMStat data contributed 57,791 datapoints outside the United States, spanning from January 1978 to March 2022. Each data point includes measurements of pH and temperature, along with concentrations of major ions and at least one redox couple. When available, redox probe measurements were also incorporated. Redox couples in the dataset encompass aqueous electron acceptors and their reduced forms (e.g., bicarbonate/methane, sulfate/sulfide, nitrate/N2, nitrate/ammonium, DO/H_2_O), as well as minerals and their reduced forms (e.g., Fe(III)/Fe^2+^ and Mn(IV)/Mn^2+^). For DO and mineral‐related redox couples, only DO, Fe^2+^, and Mn^2+^ concentrations were required.

We ensured data reliability by modeling chemical speciation and removing data points with a charge balance error (CBE) >10% (Fritz 1994). We also identified and removed outliers using the interquartile range method (Rousseeuw et al. 2006). CBE is a measure of the discrepancy between the total cations and total anions in the water sample. It is calculated from the equivalents of cations and anions (C_eq+_ and C_eq−_, respectively) according to

(3) $$\mathrm{CBE}=\frac{C_{\mathrm{eq}+}-C_{\mathrm{eq}-}}{C_{\mathrm{eq}+}+C_{\mathrm{eq}-}}\times 100\%$$

The final dataset comprises groundwater samples predominantly from three continents: North America, Europe, and Asia. These samples represent aquifers across tropical, dry, temperate, and continental climates (Cui et al. 2021). The dataset covers a variety of aquifer types, from shallow, unconfined systems to deep, confined aquifers, and includes diverse geological matrices such as sand and gravel, sandstone, carbonate, volcanic, and crystalline formations (Burow and Belitz 2014). These features ensure that the dataset is representative of different hydrological settings (Elango and Kannan 2007; Wendland et al. 2008).

### Geochemical Modeling

Chemical speciation in groundwater is simulated by using the GSS program of the Geochemist's Workbench software package (version 17.0.3) and the updated LLNL Thermodynamic Dataset (Delany and Lundeen 1990; Jin and Kirk 2018a, 2018b; Bethke 2022). GSS computes activity coefficients and accounts for non‐ideal behavior in real‐world water samples according to an extended form of the Debye‐Hückel equation, known as the B‐dot equation.

### Statistical Analysis and Model Development

We conducted Pearson correlation analyses to identify the relationships between reduction potentials and various groundwater parameters, such as pH, temperature, and measurements obtained with redox probes. Pearson correlation coefficients (*r*) and corresponding p‐values were computed using the SciPy Python package (Virtanen et al. 2020). Results with *p* < 0.05 were considered statistically significant.

To estimate the parameters of the simplified Nernst equation, we applied the Ordinary Least Squares (OLS) method. Model performance was evaluated using the Monte Carlo Cross‐Validation (MCCV) method (Haddad et al. 2013). The dataset was randomly split into a training set (80% of the data) and a test set (20%). The training set was used to calibrate the simplified Nernst equation and estimate its parameters for individual electron acceptors. The test set was used to assess model performance using metrics, such as root mean squared error (RMSE) and coefficient of determination (*R* ^2^).

This process was repeated 30 times, and the mean and standard error of the equation parameters, along with the performance matrices, were reported as their average values from the repetitions. The MCCV method reduced sampling bias and ensured diverse geographic representation in model construction (see Table S1).

## Results

### Groundwater Dataset and Geochemical Modeling

**Figure 1:**
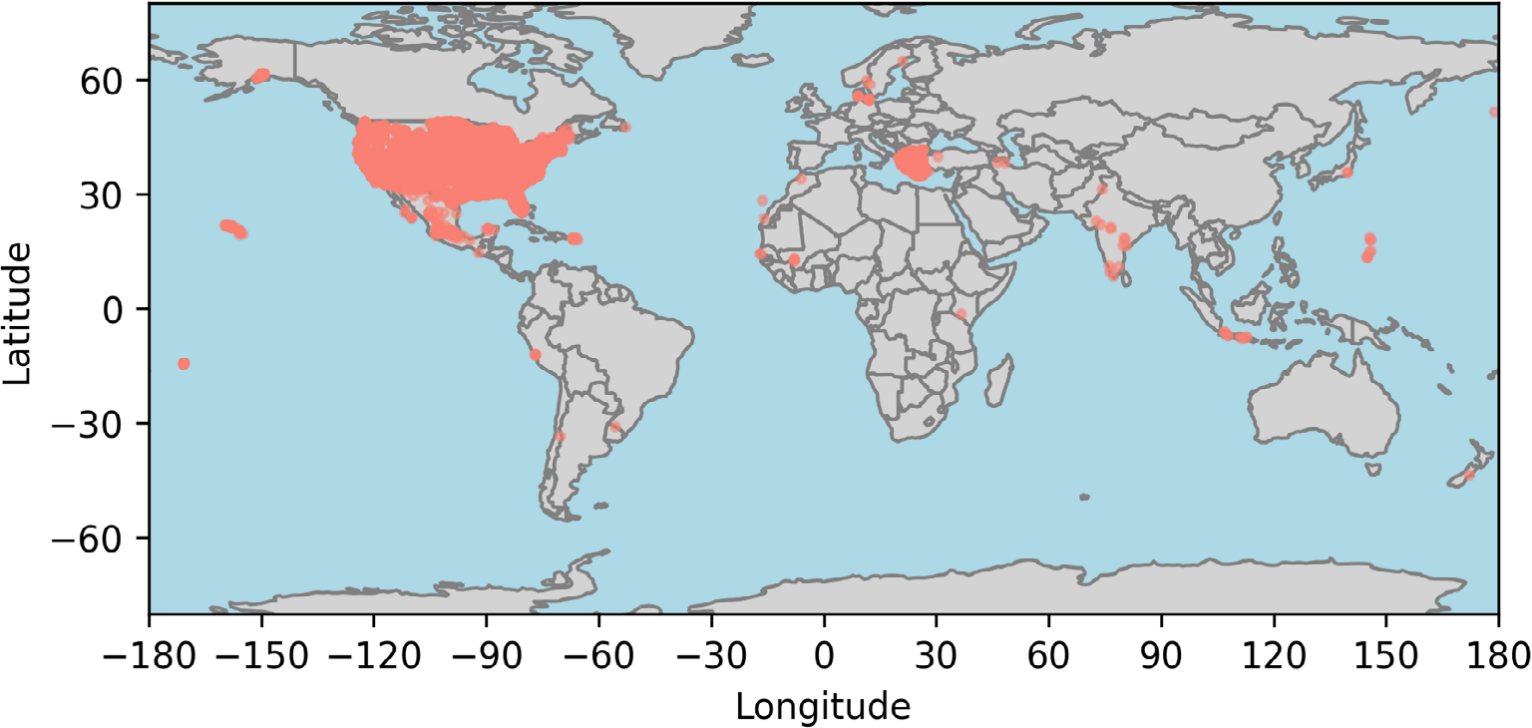
Global distribution of high‐quality groundwater chemical composition datapoints. The refined dataset includes 89,853 data points sourced from the USGS National Water Information System and GEMStat databases, representing a broad spectrum of groundwater chemical compositions and hydrological environments. Each datapoint has a charge balance error of <10%.

The final groundwater dataset includes a diverse and representative selection of groundwater samples. To ensure data quality, we removed all samples with a charge imbalance exceeding 10%, reducing the dataset to 89,853 data points (21.4% of the original dataset). High charge imbalances typically indicate analytical measurement errors or missing ionic constituents, and their exclusion ensures the reliability of chemical speciation simulations (Fritz 1994). The final dataset includes 88,124 datapoints from North America, 905 datapoints from Europe, and 726 datapoints from Asia (see Figure 1 and Materials and Methods). It encompasses a wide range of groundwater chemical compositions, reflecting major hydrochemical facies. The predominant water type is calcium‐bicarbonate (55.8% of the data points), followed by sodium‐bicarbonate (17.6%), sodium‐chloride (7.7%), magnesium‐bicarbonate (5.3%), sodium‐sulfate (4.3%), calcium‐sulfate (4.2%), and calcium‐chloride type (2.4%) (Elango and Kannan 2007). Summary statistics for pH, temperature, and specific conductivity are presented in Table 2, with additional details available in Table S2.

**Table 2:** Statistical Overview of Groundwater pH, Temperature, and Specific Conductivity.

Parameter	Mean	Median	Standard Deviation	1th Percentile	99th Percentile	25th Percentile	75th Percentile
pH	7.2	7.3	0.8	4.6	8.9	6.9	7.7
T (°C)	16.6	16.0	5.5	6.0	29.0	12.4	20.8
Specific conductivity (μS·cm^−1^)	627.5	515.0	543.7	30.0	2740.0	275.0	797.0

We amended the dataset with reduction potentials calculated with the Nernst equation for individual electron acceptors. The results of chemical speciation modeling included chemical activities of redox species, which were then applied to compute Eh values according to Equation 2. Notably, we obtained a relatively large number of Eh values for the DO/H_2_O and Mn(IV)/Mn^2+^ couples—31,871 and 38,966 values, respectively. Conversely, the number of values for the nitrate/N_2_ couple was only 20 (see Tables 1 and 3). This significant disparity in the quantities of different calculated potentials underscores the inherent bias in field analysis of groundwater redox chemistry. For instance, while our dataset includes 32,547 datapoints with measured DO levels, the total numbers of datapoints with sulfide, methane, and dissolved N_2_ concentrations are only 1209, 329, and 60, respectively.

**Table 3:** Results of Monte Carlo Cross‐Validation of the Eh‐pH‐T Equation (Equation 4) and the Eh‐pH Equation (Equation 7), Including the Redox Couples Considered, the Total Number of Data Points Used for Training and Test Datasets, the Best‐Fit Model Parameters *β*, *α*, *β*_T_, and Eh′ Obtained from the Training Sets, and the Root Mean Squared Error (RMSE) and Coefficient of Determination (*R* ^2^) Calculated for Both Equations.

Redox Couple	Dataset	*n* ^a^	Eh‐pH‐T Equation						Eh‐pH Equation				
			*α* (mV·K^−1^)		*β* (mV·K^−1^·pH^−1^)		RMSE (mV)	*R* ^2^	*β*_T_ (mV·pH^−1^)		Eh′ (mV)	RMSE (mV)	*R* ^2^
			Best‐Fit^b^	Calculated^c^	Theory^d^	Best‐Fit			Theory^e^	Best‐Fit	Best‐Fit		
DO/H_2_O	Training	20,604	−0.173 ± 0.002	−0.209 ± 0.032 (15.3%); −0.196 [−0.234, −0.182]	−0.198	−0.203 ± 0.000	9.1	0.97	−57.4	−59.8 ± 0.1	810.4 ± 0.6	11.9	0.96
	Test	5151	−^f^	−	−	−	9.1	0.97	−	−	−	11.9	0.96
${\mathrm{NO}}_{3}^{-}/N_{2}$	−^g^	20	−0.065 ± 0.045	−0.119 ± 0.025 (21.0%); −0.116 [−0.141, −0.106]	−0.238	−0.245 ± 0.006	6.7	0.99	−69.1	−74.4 ± 3.1	716.4 ± 23.8	12.3	0.97
${\mathrm{NO}}_{3}^{-}/{\mathrm{NH}}_{4}^{+}$	Training	5852	0.056 ± 0.003	0.033 ± 0.026 (78.8%); 0.039 [0.019, 0.053]	−0.248	−0.251 ± 0.000	7.5	0.99	−72.0	−73.6 ± 0.1	388.3 ± 0.9	9.3	0.98
	Test	1463	−	−	−	−	7.4	0.99	−	−	−	9.5	0.98
*β*‐MnO_2_/Mn^2+^	Training	25,831	0.581 ± 0.004	0.629 ± 0.082 (13.0%); 0.624 [0.568, 0.681]	−0.397	−0.390 ± 0.001	23.6	0.95	−115.2	−113.4 ± 0.2	609.5 ± 1.1	23.7	0.95
	Test	6458	−	−	−	−	23.6	0.95	−	−	−	23.8	0.95
*δ*‐MnO_2_/Mn^2+^	Training	25,831	0.775 ± 0.005	0.839 ± 0.109 (13.0%); 0.832 [0.757, 0.907]	−0.463	−0.454 ± 0.001	31.5	0.94	−134.3	−132.0 ± 0.2	637.4 ± 1.5	31.1	0.94
	Test	6458	−	−	−	−	31.5	0.94	−	−	−	31.3	0.94
FeOOH/Fe^2+^	Training	5345	0.998 ± 0.015	1.114 ± 0.149 (13.4%); 1.113 [1.001, 1.227]	−0.595	−0.579 ± 0.002	42.6	0.93	−172.6	−170.4 ± 0.6	−25.5 ± 4.6	44.4	0.93
	Test	1337	−	−	−	−	43.1	0.93	−	−	−	45.4	0.93
Fe(OH)_3_/Fe^2+^	Training	5345	0.998 ± 0.015	1.114 ± 0.149 (13.4%); 1.113 [1.001, 1.227]	−0.595	−0.579 ± 0.002	42.6	0.93	−172.6	−170.1 ± 0.6	49.0 ± 4.6	44.0	0.93
	Test	1337	−	−	−	−	43.1	0.93	−	−	−	45.0	0.93
Fe_2_O_3_/Fe^2+^	Training	5345	0.998 ± 0.015	1.114 ± 0.149 (13.4%); 1.113 [1.001, 1.227]	−0.595	−0.579 ± 0.002	42.6	0.93	−172.6	−170.6 ± 0.7	−105.2 ± 4.7	44.7	0.93
	Test	1337	−	−	−	−	43.1	0.93	−	−	−	45.7	0.93
${\mathrm{SO}}_{4}^{2-}/H_{2}S$	Training	588	−0.016 ± 0.014	0.063 ± 0.026 (41.3%); 0.065 [0.047, 0.080]	−0.248	−0.237 ± 0.002	7.6	0.96	−72.0	−70.6 ± 0.9	−192.4 ± 6.9	12.4	0.91
	Test	148	−	−	−	−	7.3	0.97	−	−	−	11.9	0.92
${\mathrm{HCO}}_{3}^{-}/{\mathrm{CH}}_{4}$	Training	104	0.116 ± 0.029	0.070 ± 0.029 (41.4%); 0.073 [0.046, 0.092]	−0.223	−0.229 ± 0.004	8.3	0.97	−64.7	−65.8 ± 1.3	−226.3 ± 9.9	9.9	0.96
	Test	26	−	−	−	−	7.7	0.98	−	−	−	8.7	0.97

^a^ Number of data points in dataset.

^b^ Best‐fit results are reported as mean ± standard error.

^c^ Calculated using Equation 5 from modeled chemical activities of redox chemical species; results are reported as: mean ± standard deviation (coefficient of variation); median [Q1, Q3], where Q1 and Q3 represent the 25th and 75th percentiles, respectively.

^d^ Calculated using Equation 6.

^e^ Calculated as the product of the theoretical *β* and the mean groundwater temperature (17 °C).

^f^ Not applicable.

^g^ Values estimated directly by linear regression due to a limited number of data points.

### Correlation Analyses

Considering the challenges involved in evaluating the Nernst equation (Equation 2), we sought to identify physicochemical parameters suitable for constructing a relatively simple predictive model of reduction potentials for individual redox couples. To this end, we carried out Pearson's correlation analyses between calculated Eh values (derived from geochemical modeling) and various physicochemical parameters of groundwater. To ensure practical field applicability, we focused on parameters that can be readily measured in the field, including pH, temperature, and electrode‐based redox potential.

**Figure 2:**
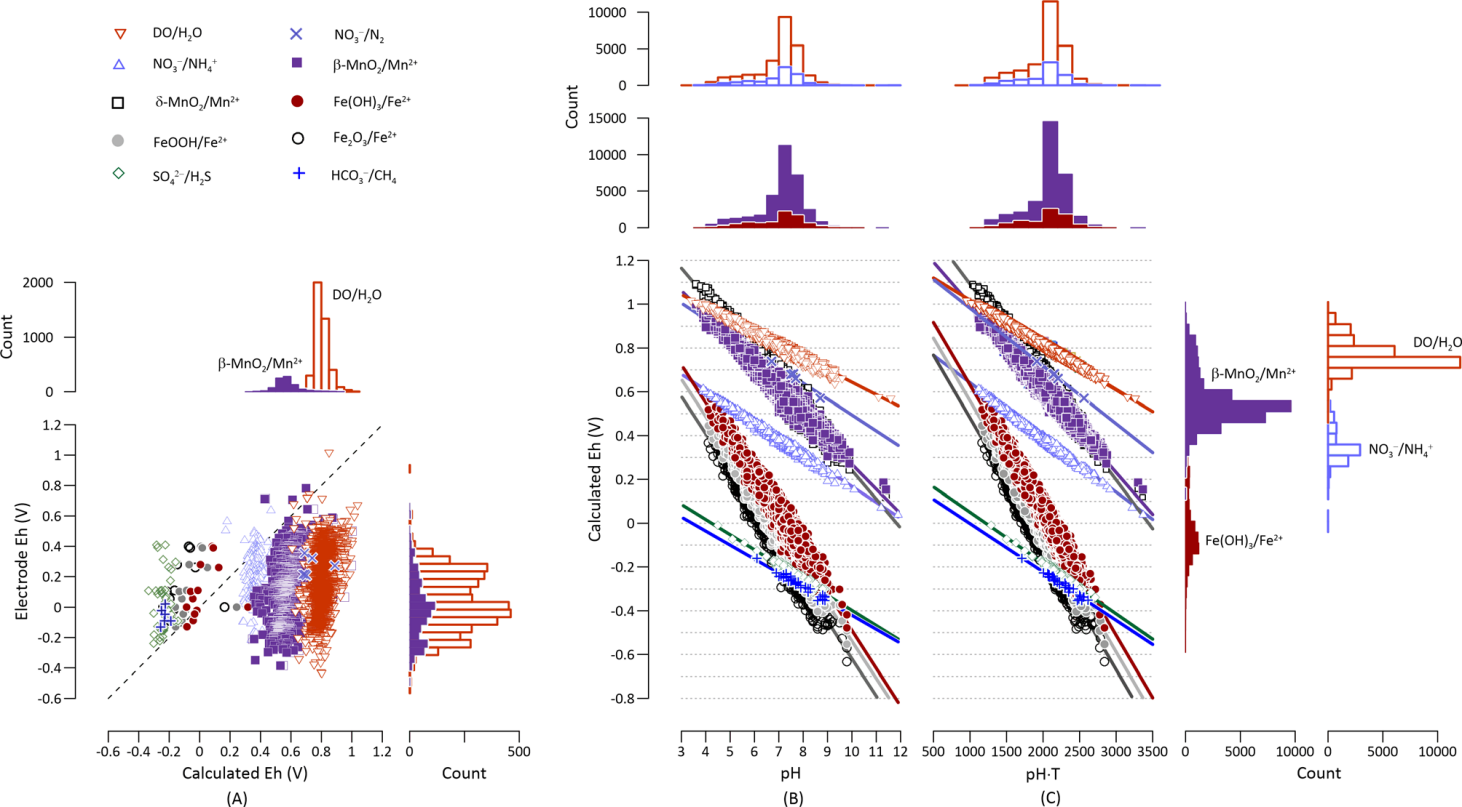
Relationships between the reduction potentials of individual redox couples calculated with the Nernst equation and field‐measured potentials, pH, and temperature. (A) Correlation between field‐measured electrode potentials and reduction potentials obtained through the Nernst equation and geochemical modeling (A). The dashed line represents the 1:1 ratio between the electrode and calculated Eh values, indicating perfect agreement. (B) Correlation between calculated Eh and pH values. The solid lines represent the linear regression fit, showing the relationship between pH and calculated Eh values. (C) Correlation between calculated Eh values and the product of pH and temperature (e.g., pH·*T*). The solid lines indicate the linear regression fit for this relationship. Histograms adjacent to each scatter plot display the distribution of data points for variables that have more than 800 observations in the dataset. Refer to Table 1 for Pearson's correlation coefficients and Table 3 for best‐fit model parameters.

Lindberg and Runnells (1984) provided the first comprehensive comparison between electrode measurements from the field and 611 calculated Eh values obtained from geochemical modeling. Here, we updated their comparison with a larger dataset consisting of 1361 field electrode measurements and 3604 modeling‐derived Eh values (Figure 2A), and evaluated the correlations between them using Pearson's correlation coefficients.

The strength of correlation between field‐measured and calculated Eh values varies across electron acceptors (see Table 1). For Mn(IV) and Fe(III) minerals, calculated Eh values show weak to modest positive correlations with electrode measurements (Pearson's *r* ≈ 0.25). In contrast, no statistically significant correlation was found for the sulfate/sulfide couple (*p* > 0.05).

Previous studies have reported close matches between electrode potentials and calculated values of the Fe(OH)_3_/Fe^2+^ couple within specific aquifer systems (Stefánsson et al. 2005; Kumar and Riyazuddin 2012). However, our broader dataset suggests that this pattern does not hold consistently across aquifers. Overall, electrode measurements tend to return lower potentials than the calculated Eh values for DO, nitrate, and Mn(IV) minerals, and higher potentials for Fe(III) minerals, sulfate, and bicarbonate.

The observed discrepancies between field electrode measurements and calculated Eh values underscore inherent shortcomings of potentiometric redox measurements (Christensen et al. 2000; Stefánsson et al. 2005; Fiedler et al. 2007). These electrodes do not selectively measure the potential of any single redox couple. Instead, they record a mixed potential resulting from simultaneous electron exchange with multiple redox species in the water. Each redox species contributes to the electrode response based on its concentration and exchange current density—a measure of its electrochemical reactivity at the electrode surface (Peiffer et al. 1992; Grundl et al. 2011). Consequently, kinetically fast and electrochemically active species tend to dominate the measured potential, even if present at low concentrations, while sensor ineffective redox species contribute less. Furthermore, the accuracy of field measurements can be compromised by electrode fouling—including interference from organic matter, biofilms, and sulfide and metal oxide coatings—as well as by exposure to atmospheric oxygen and fluctuations in environmental conditions such as pH and temperature (Nordstrom and Wilde 2005; Yalin and Shenker 2022). Collectively, these limitations challenge the application of electrode‐measured Eh values as accurate predictors of reduction potentials of specific redox couples.

Reduction potentials calculated using the Nernst equation correlate negatively with temperature, and the strength of the correlations varies across different electron acceptors (Table 1). For example, the calculated Eh values of Fe(III) minerals/Fe^2+^ have Pearson's coefficients near −0.3, whereas the corresponding values for Mn(IV) minerals/Mn^2+^ are around −0.1. These trends align with previous field observations (Prieto‐Amparán et al. 2018), and are thermodynamically justifiable based on the Nernst equation (Equation 2). This equation calculates reduction potentials by adjusting standard potentials based on environmental parameters, including temperature. The lack of strong correlation suggests that, under most groundwater conditions, the effect of temperature on calculated potentials is relatively insignificant.

Reduction potentials calculated using the Nernst equation demonstrate strong negative correlations with pH across all examined electron acceptors, with Pearson's correlation coefficients ranging between −0.96 and − 0.99 (Table 1). Additionally, these calculated potentials exhibit similarly strong negative correlations with the product of pH and temperature, with coefficients ranging from −0.97 to −0.99, slightly stronger than those observed with pH alone. These robust correlations underscore the dominant influence of pH on redox thermodynamics (Bethke et al. 2011; Jin and Kirk 2018a, 2018b). According to the Nernst equation (Equation 2), the negative correlation arises from the proton consumption in half‐cell reactions of electron acceptor reduction (Equation 1). The Nernst equation also explains the correlation between calculated Eh and the product of pH and T—temperature modulates the slope of the linear relationship between Eh and pH. These findings suggest that pH and temperature together, or pH alone, can be applied to predict the calculated potentials of the electron acceptors.

### Reduction Potential Prediction

Based on the results of correlation analyses (Table 1), reduction potentials of individual redox couples can be directly predicted from the product of pH and temperature. To enable this, we simplified the Nernst equation (Equation 2) into the following Eh‐pH‐T equation,

(4) $$\mathrm{Eh}={\mathrm{Eh}}^{o}-T\left(\alpha +\beta \mathrm{pH}\right)$$

where *α* and *β* are two parameters. The simplification is achieved by approximating the activity product *Q*_A_ with a constant, represented by the parameter *α*:

(5) $$\alpha =\frac{R}{\mathrm{zF}}\ln\left(Q_{A}\right)$$

In this formulation, *β* corresponds to the number of protons consumed per electron transfer:

(6) $$\beta =\frac{R}{\mathrm{zF}}L_{10}\nu_{H}$$

Here, *β* quantifies the sensitivity of Eh to pH at a given temperature. The product *α*·*T* quantifies the influence of the chemical activities of redox species.

We calibrated Equation 4 and assessed its performance for individual electron acceptors in aquifers by combining linear regression with Monte Carlo Cross‐Validation (MCCV) (Table 3). The calibrated Equation 4 demonstrated strong predictive accuracy, with *R* ^2^ values exceeding 0.93, indicating that >93% of the variance in calculated Eh values could be explained by groundwater pH and temperature. Moreover, the root mean squared error (RMSE) was low: deviations were <9 mV for DO, nitrate, sulfate, and bicarbonate, and <43 mV for Mn(IV) and Fe(III) minerals.

We applied the calibrated Equation 4 to predict calculated potentials in the test sets. Equation 4 exhibits robust generalization performance, maintaining similar levels of accuracy on both the training and test sets. The differences in RMSE and *R* ^2^ values between the training and test sets are <3%. Combining these results, we infer that Equation 4, together with its best‐fit *α* and *β* values, effectively predicts the calculated potentials of individual electron acceptors in aquifers.

The regression results confirm that pH is the primary control on calculated Eh, and the effects of temperature and species activity are secondary:

First, the best‐fit *β* values underscore the significant control of pH, closely matching the theoretical values calculated according to Equation 6 (see Table 3). These values indicate that a one‐unit increase in pH leads to substantial decreases in calculated potentials. At the mean groundwater temperature of 17 °C, the calculated potentials of DO, nitrate, sulfate, and bicarbonate decrease by 59 to 73 mV, while those of Mn(IV) and Fe(III) oxyhydroxides drop by 114 to 168 mV.

Second, the strong agreement between predicted Eh values from the Eh‐pH‐T equation (Equation 4) and those from full geochemical modeling supports the approximation of the activity products *Q*_A_ of redox couples as constants, that is, parameter *α* ≈ constant. This simplification is justified in part by the logarithmic form in the Nernst equation, which effectively dampens the impact of variations in species concentrations or activities.

To further evaluate this assumption, we calculated the *α* values directly from the activity product *Q*_A_ of the redox couples using Equation 5 (Table 3). For each redox couple, *α* was computed for individual groundwater datapoints and summarized using the mean, standard deviation (SD), coefficient of variation (CV), median, and interquartile range (IQR), with IQR reported as [Q1, Q3], where Q1 and Q3 denote the 25th and 75th percentiles, respectively. The median calculated *α* values closely aligned with those obtained from regression analysis. For redox couples with mean calculated *α* values exceeding ±0.1 mV·K^−1^, CVs ranged from 10% to 21%, indicating moderate but constrained variability. For couples with mean calculated *α* values between −0.1 and 0.1 mV·K^−1^, the IQR was <0.05 mV·K^−1^. Because variations in *α* (Δ*α*) propagate linearly to changes in Eh via ΔEh = Δ*α*·*T* (Equation 4), these IQR values translate to Eh fluctuations of <15 mV at the median groundwater temperature (17 °C). Together, these results support the constant *Q*_A_ approximation across the redox couples analyzed.

Lastly, the best‐fit *α* values indicate that the influence of temperature on calculated potentials is relatively limited. From the 1th and 99th percentiles of groundwater temperature (e.g., from 6 °C to 29 °C, respectively), the standard reduction potentials Eh^o^ fluctuate by no more than 11 mV. For DO, nitrate, sulfate, and bicarbonate, the products of *α* and *T* vary by <4 mV, and by 14 to 23 mV for Mn(IV) and Fe(III) oxyhydroxides. Similarly, compared to the value of *β*·*T* at 17 °C, this product changes by ~4% at 6 °C or 29 °C.

The limited temperature effect suggests a further simplification of Equation 4 by neglecting temperature variations. By treating temperature as a constant, Equation 4 reduces to the following Eh‐pH equation,

(7) $$\mathrm{Eh}=Eh^{\prime }+\beta_{T}\cdot \left(\mathrm{pH}-7\right)$$

where *β*_T_ is the product of *β* and *T* (V) and Eh′ (V) is the potential at pH 7. In line with common practice in biology, we use Eh′ as a benchmark. Notably, the Eh‐pH relationship expressed by Equation 7 is analogous to the equilibrium lines of redox couples in the Pourbaix diagram routinely constructed under the standard conditions (e.g., pH 7 and 25 °C).

Theoretical values of *β*_T_, calculated using Equation 6 and the mean groundwater temperature (17 °C), aligned closely with those derived from regression models (see Table 3). This agreement provides additional justification for treating temperature as effectively constant under typical groundwater conditions.

The performance of the Eh‐pH equation was also assessed with the MCCV method (Table 3). This equation performed consistently well across the common electron acceptors, nearly matching the predictive accuracy of the Eh‐pH‐T equation. These results support the application of the Eh‐pH equation—especially when temperature data are not available or when the temperature effect is not a primary consideration.

## Applications

The results of this study support that, in natural groundwater systems, the reduction potentials of individual redox couples can be effectively estimated using only pH and temperature, without the need for full chemical speciation modeling. This finding reinforces and quantifies the well‐established theoretical relationship between reduction potential and pH (Bethke et al. 2011; Jin and Kirk 2018a). The simplified Nernst equation (Equation 4), grounded in classical electrochemical principles and empirically calibrated (Figure 2B and 2C), enables computationally efficient integration into process‐based biogeochemical models, especially where data or computational resources are limited. This enhances the scalability of redox modeling across aquifer systems and improves our capacity to simulate redox‐sensitive processes at regional to global scales.

The simplified Nernst equation (Equation 4) and its reduced form (Equation 7), calibrated using a global groundwater dataset (Figure 2B and 2C), represent data‐driven reformulations of the classical Nernst equation (Equation 2), specifically tailored to individual electron acceptors under real‐world aquifer conditions. These formulations overcome the limitations of site‐specific geochemical modeling and relax the standard assumptions (e.g., pH 7 and 25 °C) commonly applied in traditional redox ladders and Pourbaix diagrams used for exploring thermodynamic patterns in groundwater systems (Grundl et al. 2011; Pankow 2018).

The standard redox ladder typically places O_2_ at the top, above other oxidants such as Mn(IV) oxides (Champ et al. 1979; Bethke et al. 2011; Grundl et al. 2011). However, our simplified Nernst equations reveal that this hierarchy can invert under acidic conditions. Specifically, pyrolusite and birnessite surpass O_2_ in reduction potential at pH below 3.31 ± 0.02 and 4.64 ± 0.02, respectively, making them thermodynamically more favorable electron acceptors. Notably, these minerals remain stable in acidic groundwater (see Supporting Information).

In the lower segment of the redox ladder, ferric minerals are traditionally considered stronger oxidants than sulfate and CO_2_. This arrangement is known to break down under alkaline conditions (Bethke et al. 2011). Our best‐fit simplified Nernst equations indicate that sulfate exhibits higher reduction potentials than ferrihydrite, goethite, and hematite at pH >9.34 ± 0.09, 8.60 ± 0.09, and 7.81 ± 0.09, respectively. Similarly, CO_2_ becomes a stronger electron acceptor than these ferric minerals at pH >9.61 ± 0.12, 8.89 ± 0.12, and 8.13 ± 0.12. These crossover thresholds underscore the utility of the simplified Nernst approach in identifying pH‐driven redox shifts in natural groundwater systems.

**Figure 3:**
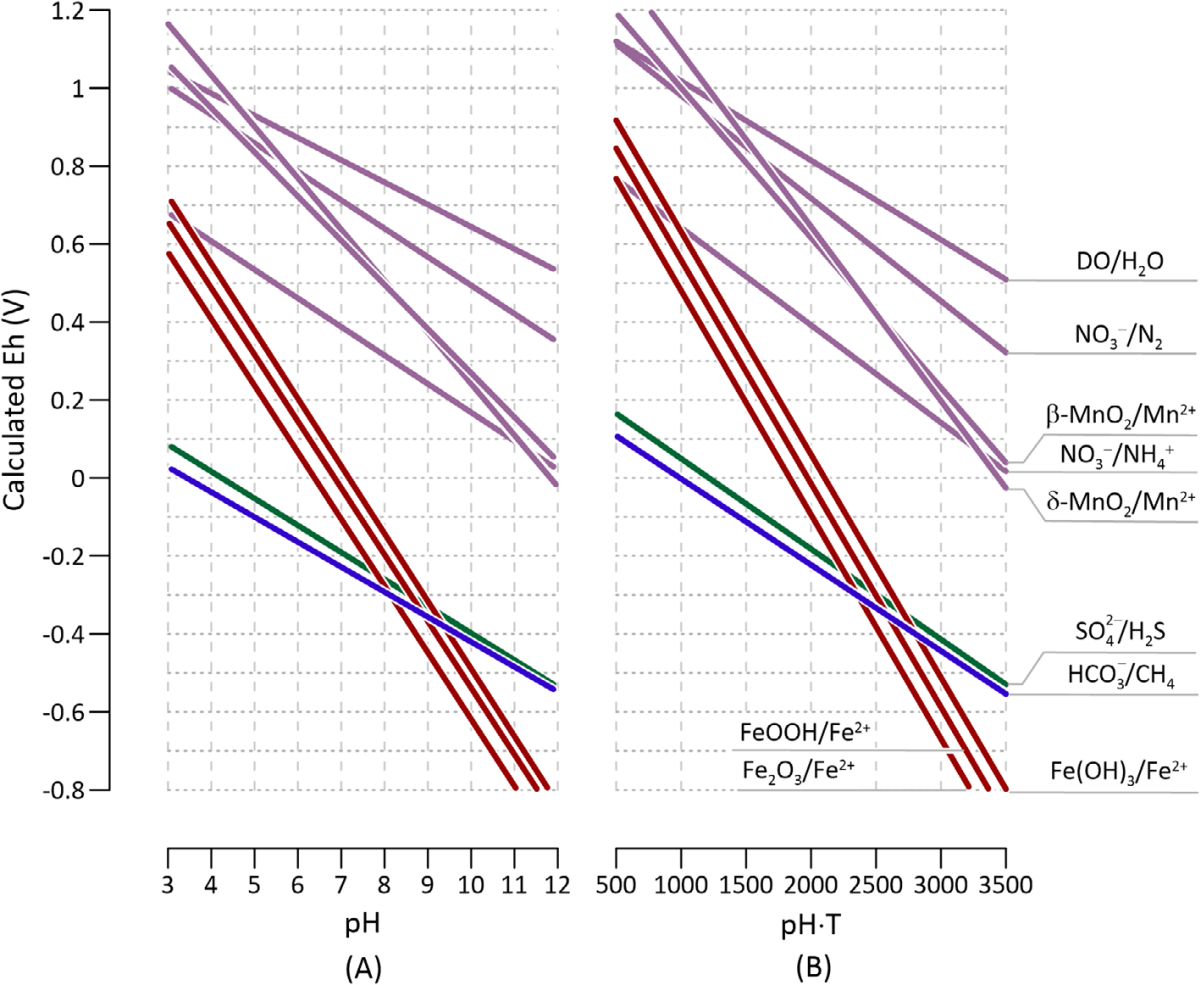
Types of anaerobic oxidation of methane (AOM) categorized based on the separation distance between the Eh‐pH equations (A) and the Eh‐pH‐T equations (B) of bicarbonate and various other electron acceptors. The three types include: (1) sulfate‐dependent AOM, (2) Fe(III)‐dependent AOM, and (3) Mn(IV)‐ and nitrate‐dependent AOM. The lines represent the best‐fit equations for the different electron acceptors, as detailed in Figure 2 and Table 3.

The simplified Nernst equation provides a simplified approach to investigate the thermodynamics of groundwater redox reactions on a broad scale, simplifying complex concepts into geometric problems. This approach can be illustrated with microbial methane oxidation (Figure 3). The thermodynamics of anaerobic methane oxidation has been evaluated with site‐specific pH values and chemical concentrations (Caldwell et al. 2008; Knittel and Boetius 2009). By leveraging the spatial relationships between the simplified Nernst equations for bicarbonate/methane and other redox couples in Figure 2B and 2C, we can extend our analysis beyond individual field sites and capture overarching thermodynamic trends of microbial methane oxidation across various aquifers. In doing so, we see that the reactions of microbial methane oxidation can be grouped into three types based on their thermodynamic states.

One type is anaerobic methane oxidation by sulfate reduction. In Figure 3, the line of sulfate/sulfide lies above the bicarbonate/methane line, which suggests that within aquifers, anaerobic methane oxidation by sulfate reduction is likely favored by thermodynamics across the entire pH range of groundwater. However, the proximity of the two lines indicates that the reaction is expected to remain close to thermodynamic equilibrium.

Another type includes methane oxidation by the reduction of ferric minerals. The intersection points between the ferric mineral and bicarbonate/methane lines suggest that methane oxidation via the reduction of ferrihydrite, goethite, and hematite becomes thermodynamically unfavorable at pH above 9.34 ± 0.09, 8.60 ± 0.09, and 7.81 ± 0.09, respectively. Consequently, these reactions are markedly influenced by pH, potentially ceasing altogether at pH levels above these thresholds. Previous experimental investigations have predominantly focused on anaerobic methane oxidation at neutral pH (Beal et al. 2009; Bar‐Or et al. 2017; He et al. 2019), and the predicted pH thresholds are yet to be tested.

The third type includes methane oxidation coupled to the reduction of Mn(IV) oxides, nitrate, and DO. These reactions are expected to stay far away from thermodynamic equilibrium, as indicated by the significant separation of the lines of these electron acceptors from the line of bicarbonate/methane.

## Discussion

This study presents a data‐calibrated simplification of the Nernst equation tailored for groundwater systems, enabling estimation of individual redox couple potentials using only pH and temperature. By isolating the thermodynamic role of pH and treating redox species activity as secondary influences, the Eh‐pH‐T equation (Equation 4) provides a scalable and computationally efficient alternative to traditional speciation‐ and modeling‐based methods.

The inclusion of both pH and temperature allows the simulation of temperature‐ and pH‐dependent feedback and forward mechanisms, such as changes in reaction kinetics, microbial activity, and mineral stability, factors crucial for accurately capturing redox dynamics in process‐based models. Where temperature data are unavailable or of secondary importance, the further simplified Eh‐pH equation (Equation 7) offers a practical approximation while retaining interpretive value.

While our model does not predict the electrode potential measured by redox probes, it offers a valuable complementary perspective. Electrode measurements reflect kinetically weighted averages of multiple redox couples in groundwater and are influenced by factors such as exchange current densities and reaction kinetics, rather than thermodynamic equilibria alone. Our approach, by contrast, isolates the reduction potential of individual redox couples, enabling more precise and comprehensive comparisons across redox zones and electron acceptors.

The data‐calibrated simplified Nernst equations offer several important advantages for groundwater research and monitoring. They enable rapid estimation of reduction potentials for individual redox couples, eliminating the need for detailed or high‐precision chemical speciation analyses. This approach is particularly well‐suited for large‐scale hydrogeological surveys, regulatory assessments, and environmental remediation efforts. In data‐sparse or resource‐limited settings, it provides a reliable and computationally efficient alternative for evaluating groundwater redox conditions.

Additionally, the simplified Nernst equations capture the overarching relationship between reduction potentials of individual redox couples and the two primary variables of groundwater—pH and temperature—across diverse aquifers. This provides fresh insights into the broad thermodynamic patterns of groundwater redox reactions beyond individual aquifers, improving the conceptual framework for understanding redox‐driven geochemical processes.

Despite its advantages, our simplified approach has certain limitations that merit further investigation. The simplified Nernst equations assume that activity products of redox couples remain relatively constant, which may not fully capture redox dynamics in systems with kinetic limitations or highly variable geochemical conditions. Additionally, while our dataset spans multiple hydrogeochemical environments, 86% of the data points originate from the United States, highlighting a geographical bias and emphasizing the need for validation using data from underrepresented regions.

In summary, this study provides a data‐driven reformulation of the Nernst equation tailored for groundwater systems, reducing the complexity of traditional speciation‐ and modeling‐based approaches while preserving thermodynamic interpretability. Our analysis demonstrates that pH is the dominant control on the reduction potential of redox couples, whereas the influences of temperature and the activity product (*Q*_A_) are consistently secondary. By integrating field‐scale observations with thermodynamic principles, the resulting Eh‐pH‐T and Eh‐pH equations offer scalable and practical tools for evaluating redox conditions in a variety of groundwater settings.

## Authors' Note

The authors do not have any conflicts of interest or financial disclosures to report.

## Supporting information


**Data S1.** Supporting Information.

## Data Availability

The data that support the findings of this study are openly available in GitHub at https://github.com/BioGeoGordon/GroundwaterRedoxPotential.
