# Supplementary material for: A Data‐Driven Simplified Nernst Equation for Estimating Reduction Potentials in Groundwater from pH and Temperature
Source: Ground Water. 2025 Aug 7;63(5):725–35. doi: 10.1111/gwat.70010 (PMC12435108; doi:10.1111/gwat.70010)
Supplement: Supplementary file 1 — Data S1. Supporting Information. [file GWAT-63-725-s001.docx]

**Supporting Information**

**A Data-Driven Simplified Nernst Equation for Estimating Reduction Potentials in Groundwater from pH and Temperature**

Gordon Bowman^§^, Gabe Harris^§^, Matthew Kirk^¶^, and Qusheng Jin^§*^

^§^Department of Earth Science, University of Oregon

^¶^Department of Geology, Kansas State University

*To whom correspondence should be addressed: [qjin@uoregon.edu](mailto:qjin@uoregon.edu), Tel: +1 (541) 346-4999; [qjin@uoregon.edu](mailto:qjin@uoregon.edu).

Number of pages: 3

Number of tables: 2

Number of figures: 1

**Table S1.** Mean and standard deviation (SD) of data point counts from North America, Asia, Europe, and Africa in the training and test datasets used in the Monte Carlo Cross-Validation (MCCV) of the Eh-pH-T and Eh-pH equations for the redox couple of O_2_/H_2_O.

**Table S2.** Statistical overview of major ion concentrations in groundwater.

**Stability of Mn(IV) minerals**

We constructed the Eh-pH diagrams for Mn(IV) minerals by using the ACT2 program of the Geochemist’s Workbench software package (version 17.0.3) and the updated LLNL Thermodynamic Dataset (Bethke, 2022; Delany & Lundeen, 1990). The results show that under oxidizing conditions, pyrolusite and birnessite remain relatively stable.


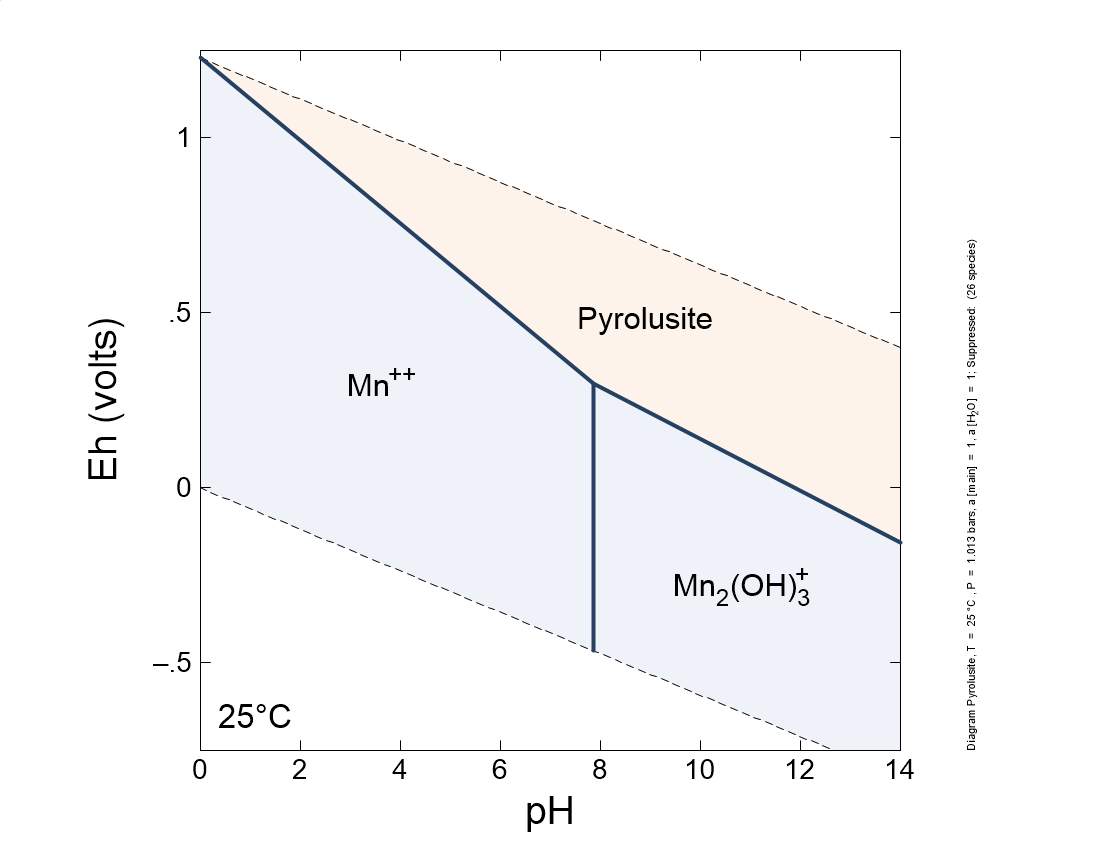

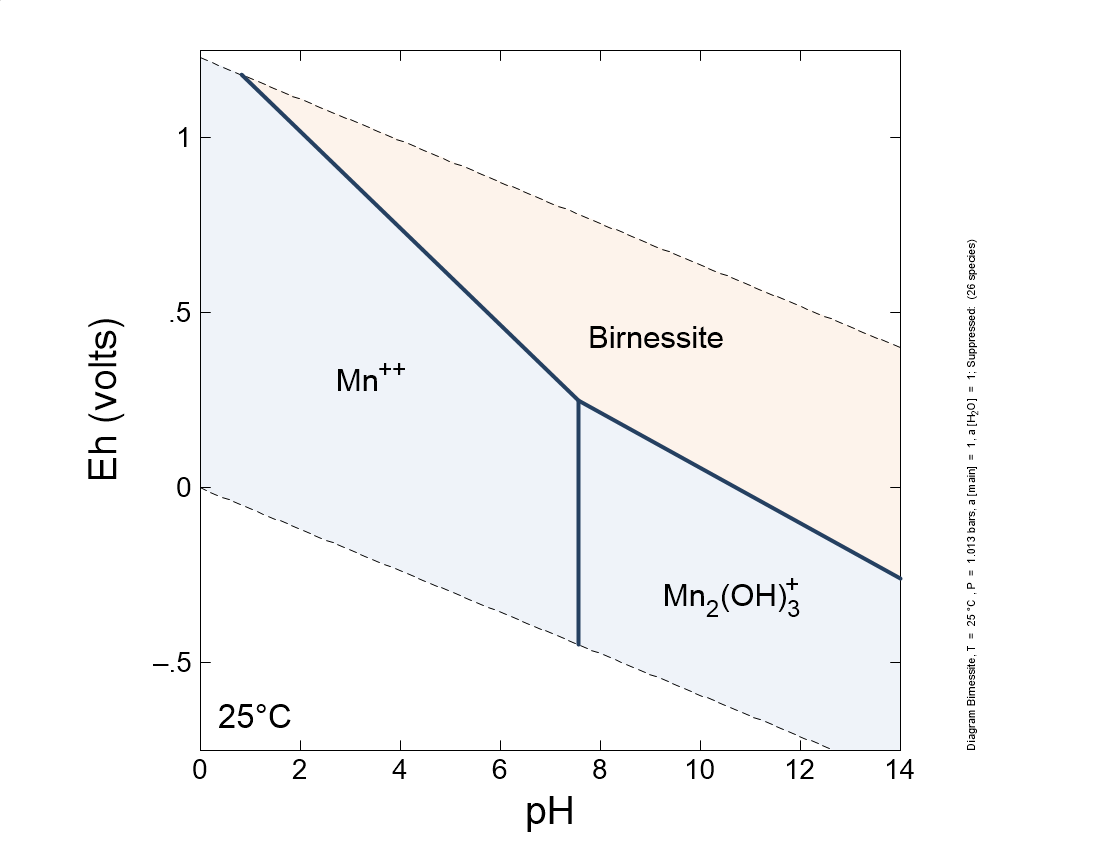


Figure S1. Eh-pH diagram for pyrolusite and birnessite. The diagrams illustrate the stability fields of the minerals and aqueous Mn(II) species over a range of redox potentials (Eh) and pH values. The boundaries between stability fields are determined based on the thermodynamic equilibrium conditions, indicating the predominant manganese species in different geochemical environments.
